# Extreme Hyponatremia Complicated by Osmotic Demyelination in a Previously Healthy Young Individual

**DOI:** 10.1177/20543581221130686

**Published:** 2022-11-15

**Authors:** Nicholas Quigley, Alexandre P. Garneau, Ludwig Haydock, Paul Isenring

**Affiliations:** 1 Nephrology Research Group, Department of Medicine, Faculty of Medicine, L’Hôtel-Dieu de Quebec du CHU de Québec, Laval University, Quebec City, Quebec, Canada; 2 Service de Néphrologie—Transplantation rénale adultes, Hôpital Necker‑Enfants malades, AP-HP, Inserm U1151, Université Paris Cité, rue de Sèvres, Paris, France

**Keywords:** extreme hyponatremia, Addison disease, osmotic demyelination

## Abstract

**Rationale:**

Severe hyponatremia can lead to dramatic complications whether it is treated or not. At times, it may be very severe (serum Na concentration: Na_S_ < 115 mmol/L) or even extreme (Na_S_ < 105 mmol/L)^a^ and its cause difficult to identify, especially in younger individuals with no history of water disorders. The case presented herein illustrates these points quite eloquently and leads us to believe that the current recommendations for the treatment of very severe hyponatremia require some fine-tuning.

**Presenting Concerns:**

A 26-year-old man was admitted to our intensive care unit for a Na_S_ of 88 mmol/L in the absence of obvious extracellular fluid volume contraction. He had been experiencing vomiting, diarrhea, fatigue, and excessive thirst for the past 6 weeks and minor neurological symptoms just before admission. Laboratory tests at presentation also showed a urine osmolarity of 697 mOsm/L and urine Na of 40 mmol/L.

**Diagnoses:**

The presenting concerns were consistent with syndrome of inappropriate antidiuretic hormone secretion (SIADH) manifesting as extreme, yet mildly symptomatic hyponatremia. At the same time, they did not point toward a specific cause initially.

**Interventions:**

The patient was treated through water restriction, subcutaneous desmopressin, and various intravenous (IV) fluids. Our goal had been to increase Na_S_ at a rate of 4 to 6 mmol/L/day and required the amount of NaCl and free water perfused hourly to be readjusted constantly. Access to water also had to be opposed as the patient was unable to tolerate his thirst.

**Outcomes:**

During the first 6 days, the rate of Na_S_ correction achieved was ~6 mmol/L/day. The patient improved initially but at the end of day 6, he experienced severe extrapontine osmotic demyelination (with widespread pyramidal and extrapyramidal deficits) that did not respond to intravenous immunoglobulin and Na_S_ relowering. A little more than 3 weeks later, he began to develop low blood pressure and a subfebrile state that revealed secondary to severe Addison disease. The water disorder and insatiable thirst subsided gradually upon replacing the deficient hormones but the neurological disorder went on to become permanent and highly disabling.

**Teaching points:**

(1) Very severe hyponatremia should always be handled as an emergency and monitored stringently in view of its potential to cause irreparable damage. (2) Because it is a major risk factor for osmotic demyelination, it should probably be corrected at a rate of less than 4 mmol/L/day especially if it is in the extreme range, chronic, or of unknown duration. (3) It can be a presenting manifestation of Addison disease.

## Abrégé

**Justification:**

Qu’elle soit traitée ou non, l’hyponatrémie grave peut entraîner des complications dramatiques. L’hyponatrémie peut être très grave (concentration de Na sérique : Na_S_ < 115 mmol/L), voire extrême (Na_S_ < 105 mmol/L)^a^, et sa cause peut être difficile à identifier, particulièrement chez les sujets plus jeunes sans antécédents de déséquilibres hydriques. Le cas présenté illustre ces points de façon éloquente et nous porte à croire que les recommandations actuelles pour le traitement de l’hyponatrémie très grave nécessitent un ajustement.

**Présentation du cas:**

Un homme de 26 ans a été admis à notre unité de soins intensifs pour une Na_S_ de 88 mmol/L sans contraction évidente du volume liquidien extracellulaire. Le patient avait souffert de vomissements, de diarrhée, de fatigue et de soif excessive au cours des six dernières semaines, et de symptômes neurologiques mineurs juste avant son admission. Les analyses de laboratoire à la présentation montraient également une osmolarité urinaire à 697 mOsm/L et une concentration de Na urinaire à 40 mmol/L.

**Diagnostic:**

Les symptômes à la présentation étaient compatibles avec un SIADH se manifestant par une hyponatrémie extrême, bien que peu symptomatique. En même temps, ces symptômes ne pointaient pas initialement vers une cause spécifique.

**Intervention:**

Le patient a été traité par restriction liquidienne, desmopressine SC et divers liquides administrés par voie intraveineuse. L’objectif était d’augmenter la Na_S_ entre 4 et 6 mmol/L/jour et il a requis que la quantité de NaCl et d’eau libre perfusée toutes les heures soit réajustée en permanence. L’accès à l’eau a également dû être restreint, car le patient était incapable de tolérer sa soif.

**Résultats:**

Au cours des six premiers jours, la correction atteinte pour la Na_S_ “était” par “a été” d’environ 6 mmol/L/jour. L’état du patient s’est d’abord amélioré, mais à la fin du 6^e^ jour, il a évolué vers une démyélinisation osmotique extrapontine sévère (avec déficits pyramidaux et extrapyramidaux étendus) qui n’a pas répondu à l’administration d’IVIG ni à la diminution de la Na_S_. Un peu plus de trois semaines plus tard, le patient a présenté une hypotension et a développé un état subfébrile qui se sont révélés secondaires à une maladie d’Addison sévère. Le déséquilibre hydrique et la soif insatiable se sont résorbés progressivement après le remplacement des hormones déficientes, mais les le désordre neurologique est devenu permanent et très invalidant.

**Enseignements tirés:**

1) L’hyponatémie très grave devrait toujours être traitée comme une urgence et surveillée de façon continue en raison de son potentiel à causer des dommages irréversibles. 2) Parce qu’elle est un facteur de risque majeur pour la démyélinisation osmotique, l’hyponatrémie devrait probablement être corrigée à un taux inférieur à 4 mmol/L/jour, surtout si elle est jugée extrême, chronique ou de durée inconnue. 3) L’hyponatrémie peut être un symptôme inaugural de la maladie d’Addison.

## Introduction

Hyponatremia, defined as a serum Na concentration (Na_S_) of 134 mmol/L or less^a^, is the most commonly encountered electrolyte abnormality in clinical practice. When it is hypotonic, it must be seen as a defect of water homeostasis that can translate into variable manifestations depending on its severity and chronicity. At times, hyponatremia can prove challenging as to its possible etiology or management. Herein, we describe such a case whose outcome was a devastating neurological complication.

## Presenting Concerns

A formerly healthy and unmedicated 26-year-old man was admitted to our intensive care unit (ICU) for extreme hyponatremia (Na_S_ of 88 mmol/L) identified in another hospital the day before. He had consulted initially for mild dysarthria, confusion, and lethargy of recent onset following a 6-week history of intermittent vomiting, diarrhea, fatigue, and intractable thirst. Before his transfer, there were no signs of focal neurological deficits or substantial extracellular fluid volume (ECFV) contraction based on physical examination.

## Clinical Findings

**Figure 1.**
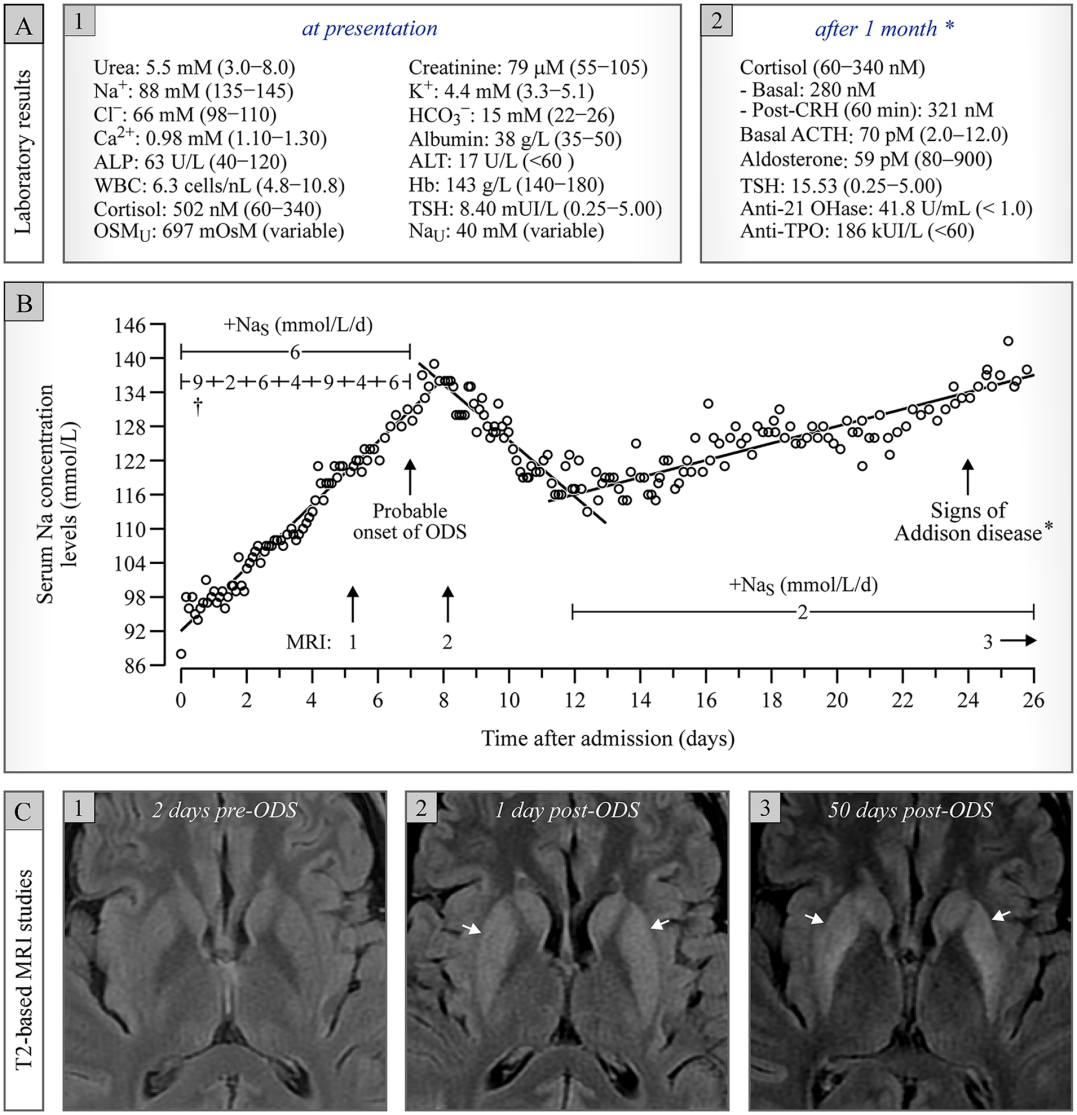
Clinical presentation: (A) laboratory results at presentation and after 1 month; (B) Na_S_ as a function of time post-admission; (C) serial T2-weighted MRI brain studies. *Note*. Basal ganglia are diffusely hyperintense on C2 and more severely so on C3 (white arrows). A small portion of the internal capsules are also hyperintense. ALP = alkaline phosphatase; WBC = white blood cell; OSM_U_ = urine osmolarity; ALT = alanine aminotransferase; TSH = thyroid-stimulating hormone; Na_U_ = urine Na; ACTH = adrenocorticotropic hormone; CRH = corticotropin-releasing hormone; OHase = hydroxylase; ODS = osmotic demyelination syndrome; MRI = magnetic resonance imaging; TPO = thyroid peroxidase. *Levothyroxine had already been started a few days postadmission for mild hypothyroidism of unknown cause initially. ^†^Thus, first value corresponds to the difference between initial Na_S_ at day 0 and mean Na_S_ for the remainder of day 1.

On arrival at our ICU, the initial clinical status was as described. In particular, blood pressure was 102/76 mm Hg with still no evidence of substantial ECFV depletion. Relevant laboratory findings at presentation and imaging features were as shown in Figure 1. Besides hyponatremia, they revealed a urine osmolarity (OSM_U_) of 697 mOsM/L, a urine Na (Na_U_) of 40 mmol/L, a slight increase in serum thyroid stimulating hormone and evening cortisol, and a normal magnetic resonance imaging (MRI) of the head on day 5 (see panels A1, B, and C1).

## Diagnostic Focus and Assessment

Based on the presenting concerns, it was concluded that the patient suffered from mildly symptomatic hyponatremia due to syndrome of inappropriate antidiuretic hormone secretion (SIADH). Whether he could have also suffered from mild ECFV contraction or been prone to excessive water intake as contributory factors could not be ruled out. At that time, the severity and etiology of hyponatremia were otherwise unclear to us.

## Therapeutic Focus and Assessment

Given that the hyponatremia was both extreme and only mildly symptomatic, it was decided to correct Na_S_ at a rate of 4 to 6 mmol/L/day. This goal was achieved by restricting water ingestion, preventing water diuresis with desmopressin (1–2 μg subcutaneously 2 or 3 times a day) and administering intravenous (IV) fluids at rates 0–150 mL/h and NaCl 0–513 mmol/L, which varied according to repeated Na_S_ measurements. Several adjustments had to be made every day, and the patient had to be physically restrained from access to water.

## Follow-up and Outcomes

During the first 6 days at the ICU, the overall clinical condition improved partially and serum HCO_3_ concentration (with 3 underscripted as written) normalized progressively. As for the rate of Na_S_ correction, it increased from 88 to 131 mmol/L over these first 6 days, that is, by a daily mean of 2 to 9 mmol/L/day for an all-around mean of ~6 mmol/L/day (see Figure 1B).

At the end of day 6, the patient became acutely stuporous while Na_S_ were all 132 mmol/L or lower and while the mean Na_S_ correction rate had increased by 5.5 mmol/L compared with the day before. A control MRI then showed that the basal ganglia had become diffusely hyperintense (Figure 1C2), consistent with extrapontine osmotic demyelination syndrome (ODS). In an attempt to reverse this condition, a single dose of 20 g intravenous immunoglobulin was administered and Na_S_ relowered with hypotonic IV fluids to an empirical target of ~115 mmol/L (Figure 1B). The patient regained awareness a few days later but developed transient akinetic mutism followed by severe and widespread pyramidal and extrapyramidal deficits.

Less than a month after the onset of ODS, he became subfebrile and hypotensive (with blood pressures of 80–100/40–70 mmHg) while affected still by intractable thirst. He was eventually found to have autoimmune polyendocrine deficiency with thyroiditis and severe Addison disease (Figure 1A2) for which he was initially treated with oral hydrocortisone (40 mg at 8 a.m., 20 mg at 12 p.m., 10 mg at 6 p.m.) and fludrocortisone (0.1 mg daily). After only 2 days under this regimen, his thirst became much less intense.

As the cause of SIADH had been elusive and was still uncertain despite the identification of an autoimmune polyendocrine disorder, additional tests were ordered including serum anti-AQP4, a focused genetic panel of water disorders^b^ and a Ga-DOTATATE-positron emission tomodensitometry scan. However, no other abnormalities could be identified, and the water disorder went completely extinct over several days even when liberal access to fluids was eventually permitted.

Although extreme^a^, hyponatremia in this case probably occurred as a result of primary adrenal failure that had begun indolently 6 weeks before admission. A Na_S_ of 88 mmol/L was not only unexpected in this setting unless associated with severe hyperglycemia. but had never been described in any other settings. In our opinion, the severity of hyponatremia was the main risk factor for the ODS and probably explains why the patient failed to recover in the long term (Figure 1C3).

## Discussion

### General Questions

This clinical vignette is that of a puzzling case of extreme hyponatremia (Na_S_ < 105 mmol/L)^a^. It took us some time to figure out why the hypotonic water disorder had developed, why this condition had been so severe, and why it had led to such a drastic complication. It is by revisiting the differential diagnosis, risk factors, and treatment of severe hypotonic hyponatremia that we were able to find answers to several of our questions.

### What was the Cause of Hyponatremia in Our Patient?

The causes of hyponatremia have been traditionally grouped into three categories based on the associated volume status (hypovolemic, euvolemic, or hypervolemic) to aid in their identification and treatment.^1^ Yet, there are multiple reasons as to why such a classification should probably not be used. In particular, it calls for potentially inadequate therapeutic measures and revolves around a clinical parameter that is notoriously difficult to assess.

**Figure 2.**
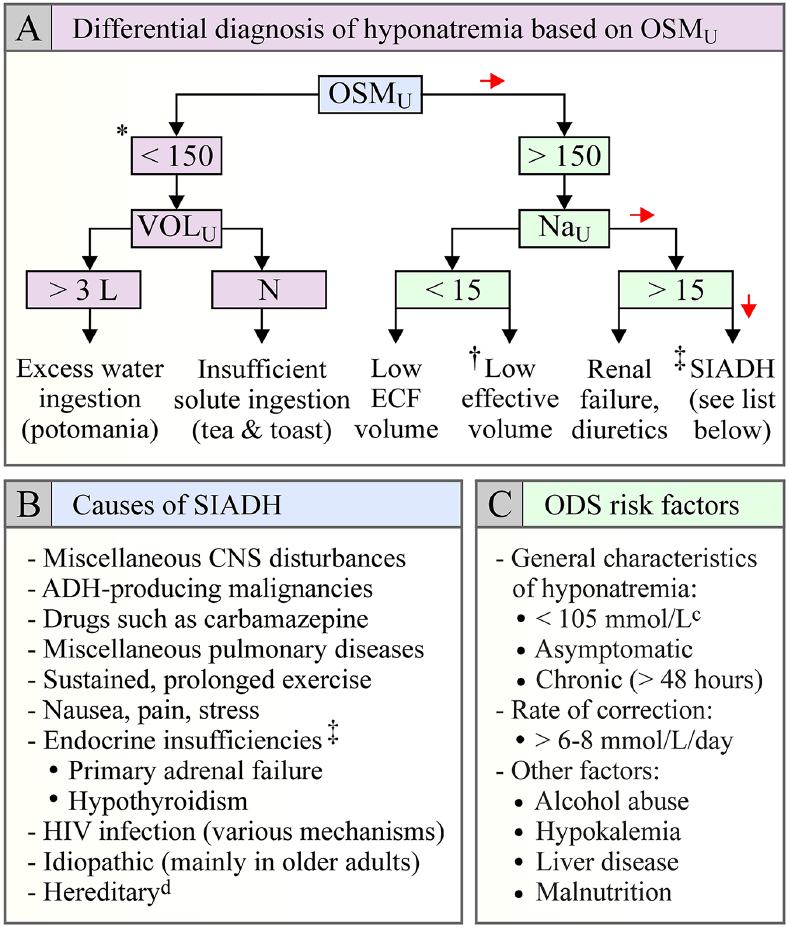
Causes of hyponatremia and risk factors for ODS: (A) causes of hyponatremia as a function of OSM_U_, Na_U_, and VOL_U_ (red arrows point toward the cause of hyponatremia in our case); (B) causes of SIADH; (C) risk factors for ODS. *Note*. Hyponatremia in Addison disease is also contributed for by urinary and gastrointestinal losses in sodium salts and by the resulting (appropriate) increase in ADH levels. OSM_U_ = urine osmolarity; VOL_U_ = urinary volume; N = normal; Na_U_ = urine Na; ECF = extracellular fluid; ODS = osmotic demyelination syndrome; CNS = central nervous system; SIADH = syndrome of inappropriate antidiuretic hormone secretion; ADH = antidiuretic hormone. *If OSM_U_ is above 150: mmol/L, renal dilution capacity is compromised, but if serum Na is very low in this context, the more OSM_U_ is close to 150: mmol/L, the more excessive water ingestion contributes to hyponatremia. ^†^Hyponatremia in heart failure and cirrhosis falls under this category. ^‡^Low serum cotisol has been found to result in corticotropin hormone-induced ADH release by parvocellular neurons and hypothyroidism to impair ADH metabolism.^4,5^

Many experts recommend classifying the causes of hyponatremia based on OSM_U_ and Na_U_ (Figure 2A) as a preferred approach to identify the mechanisms at play.^2^ In the panel shown, Addison disease and hypothyroidism may appear to have been misplaced as they are often seen as exclusion criteria for a diagnosis of SIADH.^3^ Yet, one must remember that in both endocrinopathies, antidiuretic hormone (ADH) secretion is partly inappropriate.^4,5^

Looking again at Figure 2A, it would thus appear that our patient suffered from a SIADH-type of disorder (follow red arrows) as OSM_U_ was 697 mOsm/L and Na_U_ was 40 mmol/L in the absence of renal dysfunction and diuretic treatment. Given that an exhaustive imaging exploration had also failed to reveal abnormalities aside from ODS, a limited number of etiological possibilities could have accounted for the water disorder (Figure 2B).

Among those listed, Addison disease would have certainly merited consideration early on. In particular, hyponatremia in this condition can be at times very severe^a^ with Na_S_ going down to as low as ~110 mmol/L based on a few case reports.^6^ In addition, subacute Addison disease is often difficult to diagnose in the early stages unless it is searched for through a corticotropin stimulation test.

During hospitalization, it was found that the patient did suffer from Addison disease along with thyroiditis. Although adrenal failure was not grossly apparent initially, it was still the main cause of hyponatremia in our view given that a thorough investigation failed to reveal other possibilities (see Figure 1 and Note b) and that Na_S_ normalized under hormonal therapy while water restriction could be lifted progressively.

### Why was Hyponatremia So Severe?

As far as we know, a Na_S_ of less than 90 mmol/L has never been reported before except if uncorrected for effective tonicity. This presentation was thus puzzling to us especially in the context of Addison disease. At the same time, it could have simply indicated that water homeostasis was perturbed excessively through a concomitant defect of maximal renal dilution capacity or through excessive water intake. In our case, such possibilities appeared likely based on the absence of overt Addison disease on admission.

When ADH levels are already inappropriately high regardless of the etiology at play, a decrease in effective circulatory volume or genetic predisposition is among the various factors that could further compromise renal dilution capacity. However, these factors did not appear to play a major role for our patient given that there were no obvious signs of substantial ECFV contraction at presentation and that the genetic tests failed to reveal a contributory defect.

Thiazide consumption is a well-known cause of mild hyponatremia and another factor that can compromise renal dilution capacity. From time to time, however, it can lead to severe reductions in Na_S_ as if a coexisting factor was also at work. According to one study, this cofactor could be a pre-existing tendency toward potomania,^7^ a tendency that would be well-tolerated and clinically silent until unmasked by an iatrogenic reduction in renal dilution capacity.

We believe that our patient could have thus developed extreme hyponatremia in the setting of Addison disease as he was prone to excessive water ingestion to start with. That his thirst was very important throughout most of the hospital stay would be consistent with this hypothesis. The genetic tests conducted in this regard were not conclusive but the molecular mechanisms of thirst control are still ill-defined.

### Why did the Patient Develop ODS?

The treatment of severe hyponatremia is still debated. However, the actual consensus is that Na_S_ should be increased by less than 8 mmol/L/day (and even less than 6 based on some observations)^8,9^ when it is chronic or of unknown duration^1,2,8,9^ to prevent osmolyte-poor central neuroglial cells from undergoing shrinkage-induced demyelination.^10^ In such situations, there is also consensus that the same rule should apply to very symptomatic hyponatremia but that the 6 to 8 mmol/L of increase allowed should be achieved more rapidly even if it requires Na_S_ to be kept stable for many hours afterward.

In our case, ODS occurred even if Na_S_ was increased at a mean rate of ~6 mmol/L/day and in the absence of obvious or known risk factors for ODS other than the severity of hyponatremia (see Figure 2C). For these reasons, we believe that it is the profoundness of the water disorder itself that led to the outcome observed. Although it was pretty much on par with recommendations, the rate of correction chosen could have also been simply too high in the context of a double-digit Na_S_ value.

In the light of this case, we will continue to treat very severe or extreme hyponatremia as before—through the use of water restriction, desmopressin, and various IV fluids^8-10^—while measuring Na_S_ every 2 hours during the first few days. From this moment on, however, we will try to aim for Na_S_ correction rates of 2 to 4 mmol/L/day under such circumstances.

## Conclusion

Hyponatremia of the degree experienced by our patient remains uncharted territory. It could imply that ODS is a near-inescapable aftermath. Unless hyponatremia is very symptomatic, it should probably be treated by raising Na_S_ as slowly as possible until normalization. Desmopressin should also be administered concomitantly as the treatment of hyponatremia (or of its cause) can also lead to acute ADH suppression. The same reasoning should perhaps also apply to very severe hyponatremia to be on the safe side.
